# The tree alignment problem

**DOI:** 10.1186/1471-2105-13-293

**Published:** 2012-11-09

**Authors:** Andrés Varón, Ward C Wheeler

**Affiliations:** 1 Division of Invertebrate Zoology, American Museum of Natural History, New York, NY - 10024, USA

**Keywords:** Tree alignment, Tree search, Phylogeny, Sequence alignment, Direct optimization

## Abstract

**Background:**

The inference of homologies among DNA sequences, that is, positions in multiple genomes that share a common evolutionary origin, is a crucial, yet difficult task facing biologists. Its computational counterpart is known as the multiple sequence alignment problem. There are various criteria and methods available to perform multiple sequence alignments, and among these, the minimization of the overall cost of the alignment on a phylogenetic tree is known in combinatorial optimization as the Tree Alignment Problem. This problem typically occurs as a subproblem of the Generalized Tree Alignment Problem, which looks for the tree with the lowest alignment cost among all possible trees. This is equivalent to the Maximum Parsimony problem when the input sequences are not aligned, that is, when phylogeny and alignments are simultaneously inferred.

**Results:**

For large data sets, a popular heuristic is Direct Optimization (DO). DO provides a good tradeoff between speed, scalability, and competitive scores, and is implemented in the computer program POY. All other (competitive) algorithms have greater time complexities compared to DO. Here, we introduce and present experiments a new algorithm Affine-DO to accommodate the indel (alignment gap) models commonly used in phylogenetic analysis of molecular sequence data. Affine-DO has the same time complexity as DO, but is correctly suited for the affine gap edit distance. We demonstrate its performance with more than 330,000 experimental tests. These experiments show that the solutions of Affine-DO are close to the lower bound inferred from a linear programming solution. Moreover, iterating over a solution produced using Affine-DO shows little improvement.

**Conclusions:**

Our results show that Affine-DO is likely producing near-optimal solutions, with approximations within 10% for sequences with small divergence, and within 30% for random sequences, for which Affine-DO produced the worst solutions. The Affine-DO algorithm has the necessary scalability and optimality to be a significant improvement in the real-world phylogenetic analysis of sequence data.

## Background

The inference of homologies among DNA sequences, that is, positions in multiple genomes that share a common evolutionary origin, is a crucial, yet difficult task facing biologists. Its computational counterpart is known as the multiple sequence alignment problem. There are various criteria and methods available to perform multiple sequence alignments (e.g. [1-9]). Among these, given a distance function, *to minimize the overall cost of the alignment on a phylogenetic tree* is known in combinatorial optimization as the Tree Alignment Problem (TAP) [10-15]. The TAP typically occurs as a subproblem of the Generalized Tree Alignment Problem (GTAP) which looks for the tree with the lowest alignment cost among all possible trees [10]. The GTAP is equivalent to the Maximum Parsimony problem when the input sequences are not aligned, that is, when phylogeny and alignments are simultaneously inferred.

An important element in sequence alignment and phylogenetic inference is the selection of the edit function, and in particular, the cost *G*(*k*) of a sequence of *k* consecutive insertions or deletions, generically called indels (e.g. an insertion of 3 consecutive T (*k*=3) in the sequence AA could create the sequence ATTTA. The same operation in the opposite direction would be a deletion. The sequence alignment implied would be A- - -A/ATTTA, where - represents an indel). *G*(*k*) can have a significant impact in the overall analysis [16,17]. There are four plausible indel cost functions described in the literature: *G*(*k*)=*bk* (non-affine) [18], *G*(*k*)=*a* + *bk* (affine) [18], *G*(*k*)=*a* + *b*log *k* (logarithmic) [16,19-22], and *G*(*k*)=*a* + *bk* + *c*log *k*(affine-logarithmic) [16]. Simulations and theoretical work have found evidence that affine-logarithmic yields the most satisfactory results, but provides marginal benefits over the affine function, while its time complexity is much greater [16]. For this reason, many biologists adopt the affine indel cost function. (This topic is still a subject of controversy.)

For large data sets, a popular heuristic is Direct Optimization (DO) [15]. DO provides a good tradeoff between speed, scalability, and competitive scores, and is implemented in the computer program POY [23,24]. For example, the alignment for the Sankoff *et al.* data set [11] produced by DO has cost 302.25, matching that of GESTALT [25] and SALSA [26]. Using an approximate iterative version of DO that has the same time complexity, POY finds a solution of cost 298.75, close to the best known cost of PRODALI (295.25) [27]. All other (competitive) algorithms have greater time complexities compared to DO (e.g [25-27]). An important limitation of DO, however, *is that it was not defined for affine edit distance functions*.

The properties of DO and the GTAP (DO+GTAP) for phylogenetic analysis were experimentally evaluated in [28]. The main conclusion of that study is that DO+GTAP could lead to phylogenies and alignments less accurate than those of the traditional methods (e.g. CLUSTALW + PAUP*). The initial work of Ogden and Rosenberg [28] raised a number of important questions: Do the conclusions hold if a better fit heuristic is used for the tree search in the GTAP? What would be the effect of using an affine edit distance function? How do the hypothesis scores compare among the different methods? These questions have since been answered in various followup papers.

In [29], the author found that the opposite conclusion can be drawn when a better fit heuristic for the GTAP is used. That is, when the resulting tree is closer to the optimal solution, DO+GTAP is a superior method. Moreover, a good fit heuristic is a fundamental aspect in phylogenetic analysis that cannot be overlooked.

Although [28] performed simulations under affine gap costs, the study used the non-affine distance functions described for DO at the time of publication. Whether or not a different distance function could yield different conclusions was tackled in [17]. The authors found that when using the GTAP as phylogenetic analysis criterion under the affine gap cost function, the resulting phylogenies are competitive with the most accurate method for simulated studies (i.e. Probcons using a ML analysis under RaxML) [17]. It is important to note that [17] used an early implementation of the algorithms presented in this paper (available in POY version 4 beta).

A comparison of the tree scores of various methods was recently performed in [30] and is implicit in some of the conclusions of [17]. The authors concluded that when using a heuristic fit for the GTAP, the hypotheses have scores better than those produced by other methods. Therefore, without hindsight (i.e., when accuracy cannot be measured), biologists would prefer the hypotheses generated under the GTAP.

In this paper, we introduce and present experiments for a new algorithm Affine-DO. Affine-DO has the same time complexity of DO, but is correctly suited for the affine gap edit distance. We show its performance experimentally, as implemented in POY version 4, with more than 330,000 experimental tests. These experiments show that the solutions of Affine-DO are close to the lower bound inferred from an Linear Programming (LP) solution. Moreover, iterating over a solution produced using Affine-DO has very little impact in the overall solution, a positive sign of the algorithm’s performance.

Although we build Affine-DO on top of the successful aspects of DO, DO has never been formally described, nor have its basic properties been demonstrated. To describe Affine-DO, we first formally define DO and demonstrate some of its properties.

### Related Work

The TAP is known to be NP-Hard [31]. Due to its difficulty, a number of heuristic methods are applied to produce reasonable (but most likely suboptimal) solutions. The first heuristic techniques [11,12] consist of iteratively improving the assignment of each interior vertex as a median between the sequences assigned to its three neighbors. This method can be applied to any initial assignment of sequences and adjust them to improve the overall tree cost. In recent work, Yue *et al.*[32] used this algorithm in their computer program MSAM for the tree alignment problem, using as initial assignment the median computed between the 3 closest leaves to the interior vertex (ties arbitrarily resolved).

Hein [13,14], designed a second heuristic solution which is implemented in the TreeAlign program. In TreeAlign, sets of sequences are represented by alignment graphs, which hold *all possible alignments* between a pair of sequences. The complete assignment can be performed in a post-order traversal of a rooted tree, where each vertex is assigned an alignment graph of the two closest sequences in the alignment graph of its two children vertices. The final assignment can be performed during a backtrack on the tree. Although this method is powerful, it is *not* scalable (e.g. using TreeAlign to evaluate one of the simulations used in this study did not finish within 48 hours). Moreover, the TreeAlign program does not allow the user to fully specify the distance function. This algorithm was later improved by Schwikowski and Vingron, producing the best tree alignment known for the Sankoff data set [33].

The most important theoretical results for the TAP are several 2 approximation algorithms, and a Polynomial Time Approximation Scheme (PTAS) [34-37]. These algorithms solve the TAP from a theoretical perspective, but the execution time of the PTAS renders it of no practical use. On the other hand, the 2-approximation algorithms have shown very poor performance when compared to heuristic methods such as that of TreeAlign.

Direct Optimization (DO) [15] is a heuristic implemented in the computer program POY [23,24,38], which yields good execution times and competitive alignment costs. Given that DNA sequences have a small alphabet (4 elements extended with an indel to represent insertions and deletions), DO represents a large number of sequences in a compact way by using an extended alphabet (potentially exponential in the sequence length). In the spirit of the TreeAlign method, DO heuristically assigns to each vertex, during a post order traversal, a set of sequences in an edit path connecting two of the closest sequences assigned to the child vertices. Subsequently, in a pre-order backtrack, a unique sequence is assigned to the interior vertices to produce the solution.

DO can be implemented with a time complexity of *O*(*n*^2^|*V*|), where *n* is the length of the longest sequences, and *V * is the vertex set of the tree. For larger alphabets the total time complexity is *O*(*n*^2^|*V*||*Σ*|), where |*Σ*|≪*n* is the alphabet.

Schwikowski and Vignon [27] published the best heuristic algorithm for the TAP, as implemented in the PRODALI software. Although powerful, this algorithm has a potentially exponential time and memory complexity, which in turn makes it non-scalable and difficult to use for the GTAP. Moreover, PRODALI is not publicly available. It is for these reasons that DO is the algorithm of choice for the GTAP, yielding slightly weaker tree cost approximations when compared to those of PRODALI, but suitable for better performance on larger data sets.

## Results and discussion

### Direct Optimization

Direct Optimization (DO) has only been described informally in the literature [15,38], and to build on it, we must first fill this gap. At the core of the algorithm is the use of an extended alphabet to represent sets of sequences. We begin by exploring the connection of this method with those using a tree alignment graph.

In TreeAlign and PRODALI, the set of optimal alignments between sequences are represented in an *alignment graph*. These graphs are aligned at each vertex in the tree to find their closest sequences. An alignment graph is then computed between these sequences, and assigned to a vertex of the tree. The alignment between a pair of such graphs, however, is an expensive computation, both algorithmically (*O*(*n*^4^)), and in its implementation. PRODALI is more expensive in practice, as it not only stores the set of optimal, but also suboptimal alignments.

In DO, not all the possible alignments are stored, but only one. However, it stores all the possible sequences that can be produced from this alignment. We will call such set of sequences a reduced alignment graph (RAG). Thanks to their simplicity, DO use a more compact representation of a RAG, to permit greater scalability than that of TreeAlign or PRODALI. DO represents them as sequences in an extended alphabet by which we can then represent a complete RAG with an array.

It is then possible to align RAG’s, find the closest sequences contained in them, and compute their RAG with time complexity *O*(*n*^2^). The following section formalizes these ideas.

### Sets of Sequences, Edition Distance, and Medians

**Figure 1:**
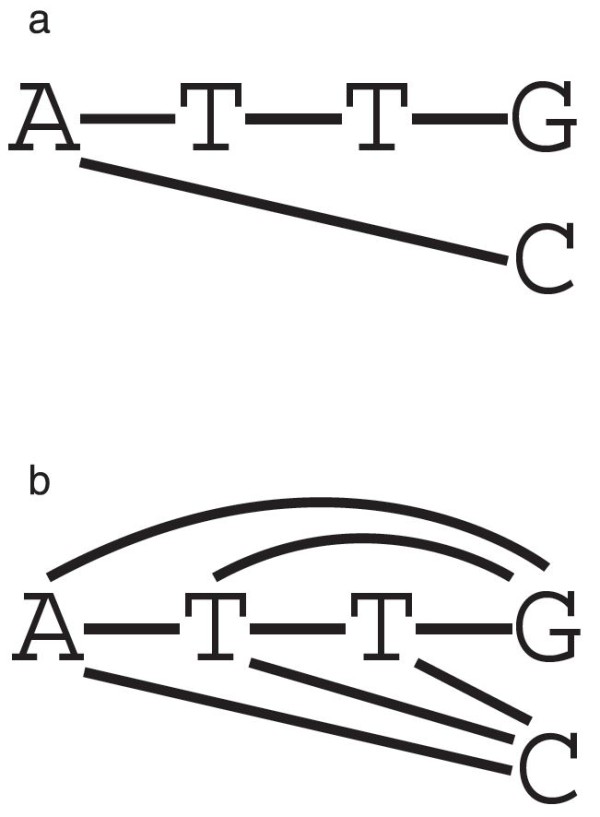
**Alignment graphs.** Graphs representing the alignment ATTG, A- -C. **a.** A plain alignment graph. **b.** An alignment graph that contains more potential sequences.

The first goal is to find a compact representation of sets of sequences produced in a pairwise alignment. For example, the alignment ATTGA--C is represented in an alignment graph shown in Figure 1. Such graph can then be extended to include intermediate sequences such as ATG or ATC (Figure 1).

The same information can be efficiently stored by using an extended alphabet $\Sigma_{P}=P(\Sigma )\setminus \{\emptyset \}$ that includes all the subsets of *Σ* with the exception the empty set, as follows

{A}{T,indel}{T,indel}{G,C}.

We call such representation a Reduced Alignment Graph (RAG). Notice that all the intermediate sequences can be produced by selecting an element from each set in the RAG, and removing all the indels from the resulting sequence. If a sequence can be generated by following this procedure, then we say that the sequence is *included* in the RAG. The example then includes the sequences ATTG, ATTC, ATC, ATG, AC, and AG.

#### Observation 1

Let $A\in \Sigma_{P}^{*}$ be a RAG. Then there are $\underset{X\in A}{\prod }|X|$ sequences contained in *A*.

In the original problem definition, we are given a distance *d* between the elements in *Σ*. Let *d*_*P*_(*A*,*B*)=min_*a*∈*A*,*b*∈*B*_*d*(*a*,*b*), be the distance between elements in *Σ*_*P*_. The following observation is by definition:

#### Observation 2

For all *A*,*B*∈*Σ*_*P*_, there exists an *a*∈*A*and *b*∈*B*such that *d*_*P*_(*A*,*B*)=*d*(*a*,*b*).

Define the RAG edit distance by setting *d*=*d*_*P*_in Equation 1.

The sequence edit distance can be computed using dynamic programming [39], following the recursive function:

(1) $$e(A_{1\ldots i},B_{1\ldots j})=\text{min}\left\{\begin{array}{c} e(A_{1\ldots i-1},B_{1\ldots j-1})+d(A_{i},B_{j})\\ e(A_{1\ldots i-1},B_{1\ldots j})+d(A_{i},\text{indel})\\ e(A_{1\ldots i},B_{1\ldots j-1})+d(B_{j},\text{indel}) \end{array}\right)$$

with base cases *e*(〈〉,〈〉)=0, and $e(\langle \rangle ,A)=e(A,\langle \rangle )=\underset{1\leq i\leq |A|}{\sum }d(A_{i},\text{indel}).$ The affine case is more elaborate but possesses the same spirit and time complexity [40].

We will show that we can find efficiently the closest sequences in a pair of RAGs, as well as their edit distance. Thanks to these properties, a RAG is used instead of an alignment graph, to bound the cost of a tree with lower time complexity.

#### Lemma 1

For all RAGs *A, B*, there exists sequences *U, V* such that *U* is contained in *A*, *V* is contained in *B*, and *e*(*A*,*B*)=*e*(*U*,*V*).

#### Proof

We define a procedure to produce *U* and *V *. Start with an empty *U* and *V *, and follow the backtrack of Equation 1. For each case, prepend the following to *U* and *V *: 

**case 1** Select an element *x*∈*X*_*i*_that holds Observation 2 and prepend it to *U*. Then find an element *y*∈*Y*_*k*_that is closest to *x* and prepend it to *V*. From Observation 2 we know that *d*(*x*,*y*)=*d*_*P*_(*X*_*i*_,*Y*_*j*_).

**case 2** Select an element *x*∈*X*_*i*_closest to *indel* and prepend *x* to *U* and *indel* to *V*. Again from Observation 2 we know that *d*(*x*,*indel*)=*d*_*P*_(*X*_*i*_,{*indel*}).

**case 3** Symmetric to case 2.

□

Observe that the overall time complexity remains *O*(*n*^2^) as in the original Needleman-Wunsch algorithm [39].

### The DO Algorithm

### *DO*(*T*,*χ*), Direct Optimization

DO (Algorithm *DO*(*T*,*χ*), Direct Optimization) estimates the cost of a tree by proceeding in a post-order traversal on a *rooted tree*, starting at the root *ρ*, and assigning a RAG to each interior vertex. 

**Data**: A binary tree *T* with root *ρ*

**Data**: An assignment *χ*:*L*(*T*)→*Σ*of sequences to the leaves *L* of *T*

**Data**: *S*(*v*) holds a set of sequences for vertex *v*, initially empty for every vertex

**Result**: *cost* holds an upper bound of the cost of *T*,*χ*

begin

*cost*←0

**foreach***level of T, with the bottom level first***do**

**foreach***node v at the level***do**

**if***v* is a leaf (has no children) **then**

*S*(*v*)←〈*a*_*i*_,*a*_*i*_={*χ*(*v*)_*i*_}〉

else

**Data**: *v* has children *u* and *w*

*cost*←*cost* + *e*_*P*_(*S*(*u*),*S*(*w*);

*U*,*W*← the alignment of *S*(*u*) and *S*(*w*)) respectively;

*S*(*v*)←*m*_*P*_(*U*,*W*);

end

end

**return***cost*

end

end

We have not defined yet *m*_*P*_(*U*,*W*) to compute each RAG. Let *m*(*X*,*Y*)*m*(*X*,*Y*) be the set of elements in *X* and *Y * that realize the distance *d*_*P*_(*X*,*Y*). Let the RAG between two aligned RAGs $A,B\in \Sigma_{P}^{*},|A|=|B|=n$ be

$$m_{P}(A,B)=\langle x_{i}=m(A_{i},B_{i})\rangle .$$

Without loss of generality, assume from now on that for all *x*∈*Σ*∖{*indel*}, *d*(*x*,*indel*)=*b* for some constant *b*.

#### Lemma 2

Let *C*=*m*_*P*_(*A*,*B*). Then for all *X* included in *C*, there exists *Y* and *Z* included in *A* and *B* respectively, such that *e*_*P*_(*A*,*B*)=*e*(*Y*,*Z*)=*e*(*X*,*Y*) + *e*(*X*,*Z*)*.*Moreover, *Y* and *Z* are (some of) the closest sequences to *C* that are contained in *A* and *B* respectively.

#### Proof

Follows directly from the median definition and Lemma 1. □

Lemma 2 is important for the correctness of the DO algorithm. It shows that for every sequence contained in *C*, there are corresponding sequences in *A* and *B* of edit distance equal to *e*_*P*_(*A*,*B*). This lemma can then be used in the DO algorithm to delay the selection of a sequence from each RAG, and use *e*_*P*_ directly to calculate the overall cost of the tree. Without it, *e*_*P*_cannot be used for this purpose directly.

#### Definition 1

Compatible assignments Two assignments *χ*:*V*→*Σ*^∗^and *χ*^*′*^:*V*→*Σ*^∗^are *compatible* if both assign the same sequences to corresponding leaves, that is, for all *v*∈*L*, *χ*(*v*)=*χ*^*′*^(*v*).

The following Theorem shows that the tree cost computed by DO is feasible:

#### Theorem 1

There exists an assignment of sequences *χ*^*′*^compatible with *χ*such that

$$\text{DO}(T,\chi )=\underset{(u,v)\in E}{\sum }e(\chi^{\prime }(u),\chi^{\prime }(v)).$$

#### Proof

Let *T* have root vertex *ρ*. Call *χ*^*′*^the final assignment of sequences to the vertices of *T*. Select any *X* included in *S*(*ρ*) and set *χ*^*′*^(*ρ*)←*X*. Then for each other vertex *v* with parent *p*, following a pre-order traversal starting at *ρ*, let *χ*(*v*)←*X*where *X*∈*Σ*^∗^is included in *S*(*v*) and is closest to *χ*^*′*^(*p*). From Lemma 2, we know that for any selection at *p* there exists a selection in its children that would yield the additional cost computed at *p* during the DO algorithm. Moreover, at each pre-order traversal step, we assign to each vertex *v* the closest sequence to *χ*^*′*^(*p*) included in *S*(*v*). Again from Lemma 2, we know that the total cost of the two edges connecting *p* with its children must be greater than or equal to the additional cost computed for vertex *p* in the DO algorithm. Therefore, $\text{DO}(T,\chi )\geq \underset{\left(u,v)\in E(T\right)}{\sum }e(\chi^{\prime }(u),\chi^{\prime }(v))$. □

DO is weaker than the alignment graph algorithms [14,27,33], as the later techniques maintain the set optimal edit paths between sequences, or a superset including it. However, in these algorithms the overall execution time and memory consumption requirements could grow exponentially [27]. In contrast, DO maintains a polynomial memory and execution time, making it more scalable, with competitive tree scores. Moreover, DO can be efficiently implemented thanks to the simplicity of the data structures involved.

### The Affine Gap Cost Case

**Figure 2:**
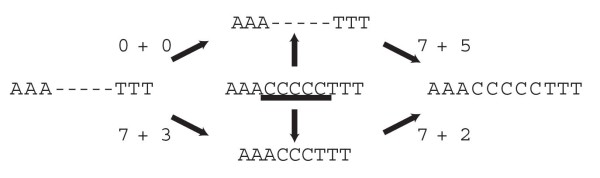
**Example of suboptimal median.** Let *G*(*k*)=7 + *k*. The center sequence is the median for the alignment of the left and right sequences. (The underscored C represents {*C*,*indel*}.) Although the upper and lower sequences are included in the median, the lower one is not in an optimal edit path connecting left and right. This example shows Lemma 2 does not hold for affine gap costs. Therefore, there are sequences in this RAG that cannot be used directly in the DO algorithm without an extra cost, not computed by *e*_*P*_. It follows that DO, if used directly for the affine gap cost case, can compute an incorrect cost for a given tree.

In practice, biologists use DO because of its scalability and competitive costs. However, the DO algorithm was defined for the non-affine distance functions (*G*(*k*)=*bk*), and does not work correctly for the popular affine indel cost model [18] (*G*(*k*)=*a* + *bk*). Under many parameter sets, DO could produce worse tree cost estimations than those of the Lifted Assignment if used under the affine gap cost model (non published data). The fundamental reason for this problem is that Lemma 2 does not hold for the affine gap cost (e.g. Figure 2), and therefore, *e*_*P*_ cannot be directly used to correctly bound the cost of a tree.

To overcome this problem, we extend Gotoh’s algorithm [40] to compute distances heuristically for sequences in $\Sigma_{P}^{*}$, and define a new median sequence. With these tools, we modify DO so that Lemma 2 still holds to compute tree cost bounds.

#### Heuristic Pairwise RAG Alignment

Let *A* and *B* be a pair of RAG’s to be aligned. Define the affine edit distance function, analogous to *e*_*P*_, using 4 auxiliary matrices (*g*,*d*,*v*, and *h*), as

$$e_{{\text{aff}}_{P}}(A_{1\ldots i},B_{1\ldots j})=\text{min}\;\{g[i,j],d[i,j],v[i,j],h[i,j]\}.$$

The matrices *g*,*d*,*v*, and *h* will be filled recursively. Before defining them formally, the basic intuition of the procedure is that *g*[*i*,*j*] is the cost of an alignment where *A*_*i*_ and *B*_*j*_ align elements other than an *indel*. *d*[*i*,*j*] is the cost of an alignment using indel elements in *A*_*i*_and *B*_*j*_. *v*[*i*,*j*] is the cost of an alignment where we use a “vertical” indel block by aligning *B*_*j*_ with an indel. Finally, *h*[*i*,*j*] is the cost of an alignment where we use a “horizontal” indel block by aligning *A*_*i*_ with an indel.

To compute these values, we define a number of accessory functions. The cost of a pure substitution *subst*(*X*,*Y*)=*d*_*P*_(*X*∖{*indel*},*Y*∖{*indel*}). Symmetric to the substitution cost, we need the cost of *extending* a gap when indel∈A,B⊆Σ:

$$\begin{array}{lll} \text{diag}(X,Y) & = & \left\{\begin{array}{l} 0\;\text{if}\;\;\text{indel}\in X\text{and}\text{indel}\in Y\\ \infty \;\text{otherwise.} \end{array}\right) \end{array}$$

There are three remaining accessory functions required to compute the matrices *g*,*h*,*v*, and *d*. Each function handles various cases where *a* or *b* needs to be added. The first function, *go*(*A*,*i*) evaluates whether or not it is necessary to add a gap opening value when aligning *A*_*i*_ with a gap:

$$\begin{array}{lll} \text{go}(A,i) & = & \left\{\begin{array}{l} 0\;\text{if}\;\;i=1\;\;\text{and}\;\;\text{indel}\in A_{i}\\ 0\;\text{if}\;\;i>1\;\;\text{and}\;\;\text{indel}\notin A_{i-1}\text{and}\;\;\text{indel}\in A_{i}\\ a\;\text{otherwise.} \end{array}\right) \end{array}$$

The second function *go*^*′*^(*X*,*Y*) calculates the extra cost incurred when *not* selecting an indel in one of the sequences means splitting an indel block:

$$\begin{array}{lll} go^{\prime }(X,Y) & = & \text{subst}(X,Y)+\left\{\begin{array}{l} 0\;\text{if}\;\;\text{indel}\notin X\\ a\;\text{otherwise.} \end{array}\right) \end{array}$$

The third, and final accessory function, computes what would be the extra cost of *extending* an indel, that is:

$$\begin{array}{lll} \text{ge}(X) & = & \left\{\begin{array}{l} 0\;\text{if}\;\;\text{indel}\in X\\ b\;\text{otherwise.} \end{array}\right) \end{array}$$

Finally, the recursive functions for the cost matrices is defined as:

(2) $$\begin{array}{lll} g[i,j] & = & \text{min}\;\left\{\begin{array}{l} g[i-1,j-1]+\text{subst}(A_{i},B_{j})\\ d[i-1,j-1]+\text{subst}(A_{i},B_{j})+\\ \;\text{go}(A,i)+\text{go}(B,j)\\ v[i-1,j-1]+go^{\prime }(B_{j},A_{i})\\ h[i-1,j-1]+go^{\prime }(A_{i},B_{j}), \end{array}\right) \end{array}$$

(3) $$\begin{array}{lll} h[i,j] & = & \text{min}\;\left\{\begin{array}{l} h[i,j-1]+\text{ge}(B_{j})\\ d[i,j-1]+\text{ge}(B_{j})+\text{go}(B,j), \end{array}\right) \end{array}$$

(4) $$\begin{array}{lll} v[i,j] & = & \text{min}\;\left\{\begin{array}{l} v[i-1,j]+\text{ge}(A_{i})\\ d[i-1,j]+\text{ge}(A_{i})+\text{go}(A,i), \end{array}\right) \end{array}$$

(5) $$\begin{array}{l} d[i,j]=\text{diag}(A_{i},B_{i})+ \end{array}$$

(6) $$\begin{array}{lll} & & \text{min}\;\left\{\begin{array}{l} d[i-1,j-1]\\ g[i-1,j-1]+\text{go}(A,i)+\text{go}(B,j), \end{array}\right) \end{array}$$

with base cases *g*[0,0]=0,*d*[0,0]=*∞*,*v*[0,0]=*go*(*A*,1), *h*[0,0] = *go*(*B*,1),*g *=[0,*i*]= *d*[0,*i*]= *v*[0,*i*] =*∞*,*h*[0,*i*]= *h*[0,*i*−1] + *ge*(*B*_*i*_),1≤*i*≤|*B*|,*v*[*j*,0]=*v*[*j*−1,0] + *ge*(*A*_*j*_), and *g*[*j*,0]=*d*[*j*,0]=*h*[*j*,0]=*∞*,1≤*j*≤|*A*|.

The following theorem shows that if we align a pair of sequences in *A*,*B*, then we can bound the cost of the closest pair of sequences included in them.

##### Theorem 2

There exists a sequence *X* contained in *A* and a sequence *Y* contained in *B* such that $\left(e_{{\text{aff}}_{P}}(A,B)\geq e_{\text{aff}}(X,Y)\right)$.

##### Proof

We are going to create a pair of sequences *X* and *Y * contained in *A* and *B* respectively that have edit cost at most $\left(e_{{\text{aff}}_{P}}(A,B)\right)$. To do so, follow the backtrack that yields $\left(e_{{\text{aff}}_{P}}(A,B)\right)$, and at each position *i* and *j* in the aligned *A* and *B* assign *X*_*k*_and *Y*_*k*_, where *k* is the alignment position corresponding to the aligned *X*_*i*_and *Y*_*j*_as follows: 

1. *g*[*i*,*j*] is the cost of aligning *A*_1…*i*_and *B*_1…*j*_when a non-indel element of *A*_*i*_and *B*_*j*_is aligned. If the backtrack uses *g*[*i*,*j*] then assign to *X*_*i*_and *Y*_*j*_the closest elements in *A*_*i*_∖*indel*and *B*_*j*_∖*indel*. Observe that all the cases in Equation 2 align a non-indel element from *A*_*i*_and *B*_*j*_, and add a cost that is always greater than or equal to *subst*(*A*_*i*_,*B*_*j*_)=*d*(*X*_*i*_,*Y*_*j*_).

2. *h*[*i*,*j*] is the cost of extending an indel in the horizontal direction. Therefore, select *X*_*k*_=*indel*, and

$$Y_{k}=\left\{\begin{array}{c} \text{indel}\;\;\;\text{if}\;\;\text{indel}\in B_{j}\\ y,y\in B_{j}\;\text{otherwise}. \end{array}\right)$$

If *Y*_*k*_=*indel*, then the alignment of *X*_*k*_and *Y*_*k*_causes no additional cost in the particular alignment being built between *X* and *Y*. Otherwise, then there is an extra cost, of at least the *b* parameter, which both cases of Equation 3 account for. Additionally, if the previous pair of aligned elements are a pair of indels (second case in 3, see below for the treatment of this option), then an extra indel opening cost is added.

3. *v*[*i*,*j*] is the cost of extending an indel block in the vertical direction. The treatment is symmetric to that of *h*, with *Y*_*k*_=*indel*and

$$X_{k}=\left\{\begin{array}{c} \text{indel}\;\;\;\text{if}\;\;\text{indel}\in A_{i}\\ x,x\in A_{i}\;\text{otherwise}. \end{array}\right)$$

4. *d*[*i*,*j*] is the cost of extending an indel in the *diagonal direction*, that is, when both *A* and *B* hold indels, and those indels are being selected during the backtrack. Equation 6 ensures that this choice is only possible by assigning *∞* whenever at least one of *A*_*i*_or *B*_*j*_does not contain an indel. Otherwise, if this option is selected, then simply assign *indel* to both *X*_*k*_ and *Y*_*k*_with no extra cost for the alignment of *X* and *Y*.

□

#### The Main Algorithm: Affine-DO

We will now use $\left(e_{{\text{aff}}_{P}}(A,B)\right)$ to bound the cost of a tree using a post-order traversal, in the same way we did with DO (Algorithm *DO*(*T*,*χ*), Direct Optimization). In order to do so a RAG to be assigned on each step must be defined (i.e. the function *m*_*P*_in Algorithm *DO*(*T*,*χ*), Direct Optimization). To create the RAG *M* (initially empty), do as follows in each of the 4 items described in the proof of Theorem 2: 

1. If we selected two indels in *X*_*k*_and *Y*_*k*_, don’t change *M*.

2. If *X*_*k*_=*indel*and *Y*_*k*_≠*indel*, then prepend $\{\text{indel}\}\cup B_{j}$ to *M*.

3. If *X*_*k*_≠*indel*and *Y*_*k*_=*indel*, then prepend $\{\text{indel}\}\cup A_{i}$ to *M*.

4. If *X*_*k*_≠*indel*and *Y*_*k*_≠*indel*, then prepend {*x*∈*A*_*i*_,for some *y*∈*B*_*j*_,*d*(*x*,*y*)=*d*(*X*_*k*_,*Y*_*k*_)} + {*y*∈*B*_*j*_,for some *x*∈*A*_*i*_,*d*(*x*,*y*)=*d*(*X*_*k*_,*Y*_*k*_)} to *M*.

5. Once the complete *M*^*′*^is created, remove all the elements *M*_*i*_={*indel*} to create the indel-less RAG *M*. We call *M* the RAG produced by $\left(m_{{\text{aff}}_{P}}(A,B)\right)$.

##### Definition 2

Affine-DO Affine-DO is Algorithm *DO*(*T*,*χ*), Direct Optimization, modified by replacing *m*_*P*_with $\left(m_{{\text{aff}}_{P}}\right)$, and *e*_*P*_with $\left(e_{{\text{aff}}_{P}}\right)$.

It is now possible to use the Affine-DO algorithm to bound heuristically the cost of an instance of the TAP.

##### Theorem 3

Given a rooted tree *T* with root *ρ*, and an Affine-DO assignment $S:V(T)\to \Sigma_{P}^{*}$, there exists an assignment *χ*^*′*^:*V*(*T*)→*Σ*^∗^such that *X*=*χ*^*′*^(*ρ*) and the cost computed by Affine-DO equals that implied by *χ*^*′*^.

##### Proof

If there are no indels involved in the tree alignment, then the arguments of Theorem 1 would suffice. Hence, we now concentrate on the cases that involve indels.

**Figure 3:**
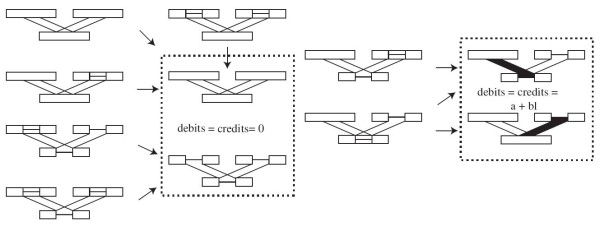
**Credits and debits in the simple cases.***credits* and *debits* incurred by the different possible arrangements of subsegments with matching limits in *S*(*p*), *S*(*u*), and *S*(*v*). The only cases with *credits*=*debits*>0 (in the right box) represents with filled boxes the assignments that would yield an indel block.

**Figure 4:**
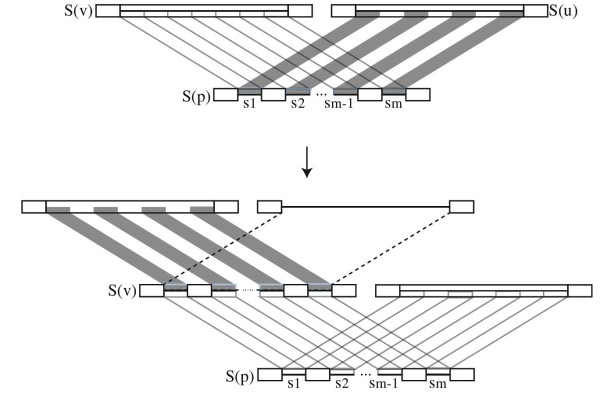
**Credits and debits in the complex cases.** In the upper part, overlapping blocks of type B in *S*(*u*) and *S*(*v*), with a complex pattern of insertions and deletions in *S*(*p*). The total *credits* added at *S*(*p*) by Affine-DO can be transferred to *u* and *v*. In the lower, the credits transferred to *v* can be assigned to *m* individual insertion blocks (solid boxes), and one deletion block (dashed empty box) which maintain *debits*>*credits* .

To prove those remaining cases, we will use induction on the vertices of the tree. To do so, we will count the *credits* that each vertex adds to the subtree it roots as added by the Affine-DO algorithm. The credits represent the maximum total cost of the indels involved in a particular subtree; we will compare them with the *debits* incurred by a set of indels, and verify that the *credits* are always greater than or equal to the *debits*. To simplify the description, we will call type A subsequences of maximal size holding only indels, and type B subsequences of maximal size holding sets that include, but are not limited to, indels, and type C maximal subsequences holding sets with no indel. We will count without loss of generality the *credits* and *debits* within those subsequences. In Figures 3 and 4, Type A is represented as a line, type B as a box with a center line, and type C as an empty box.

For the inductive step, consider the leaves of the tree. By definition, for all *v*∈*L*, *S*(*v*) can contain subsequences of neither type A nor B, as there are no indels allowed. Therefore, the theorem holds true, with a *credits*=*debits*=0.

Consider now the interior vertex *v*, with children *u* and *v*. In Figure 3, all the simple cases where the limits of the subsequences in *S*(*u*) and *S*(*v*) match those of *S*(*p*). It is straightforward to see that in all those cases *credits*=*debits*.

Consider now the more difficult case when the blocks do not have exact limits. Assume without loss of generality that *S*(*u*) and *S*(*v*) have a segment of type B, and *S*(*p*) has in the corresponding segment a series of blocks of type A and C (Figure 4). (There can be no subsequences of type B in *S*(*p*) aligned with those of type *B* in *S*(*u*) and *S*(*v*) as $\left(m_{{\text{aff}}_{P}}\right)$ does not allow it.)

The total credit granted by Equation 2 is $c\geq 2\mathrm{ma}+2b\overset{m}{\underset{i=1}{\sum }}s_{i}$. We can transfer *c*/2 to *u* (*v*), so that in one edge rooted by *u* (*v*), a series of insertions corresponding to the subsequences *s*_1_,*s*_2_,…,*s*_*m*_can occur (Figure 4), while the other branch supports a single deletion of length $l-\overset{m}{\underset{i=1}{\sum }}s_{i}$ (Figure 4 lower, upper dashed box). The total debit of these events now rooted in *u* would be

(7) $$a(m+1)+b\overset{m}{\underset{i=1}{\sum }}s_{i}+b(l-\overset{m}{\underset{i=1}{\sum }}s_{i})\leq c/2+a+\mathrm{bl.}$$

By the inductive hypothesis, the subtree rooted by *u* (*v*) has *credits*≥*debits*, and from Equation 7 we also have that *credits*>*debits*in *p*, therefore the theorem holds, and the overall tree rooted by *p* has a sequence assignment of cost at most that computed by the Affine-DO algorithm. □

##### Theorem 4

If *Σ*is small, then Affine-DO has time complexity *O*(*n*^2^|*V*|) time, otherwise the time complexity is *O*(*n*^2^|*V*||*Σ*|).

##### Proof

If the alphabet is small, then $\left(m_{{\text{aff}}_{P}}\right)$ and *d*_*P*_can be pre-computed in a lookup table for constant time comparison of the sets. For large alphabets the maximum size of the sets contained in *Σ*_*P*_can be made constant. Otherwise, a binary tree representation of the sets would be necessary, adding a |*Σ*| factor to the set comparison. Each heuristic alignment can be performed using dynamic programming, with time complexity *O*(*n*^2^) where *n* is the maximum sequence length (Ukkonen’s [41] algorithm makes no obvious improvement as insertions and deletions could have cost 0 when aligning sequences in $\Sigma_{P}^{*}$). Each alignment must be repeated for |*V*| vertices during the post-order traversal, yielding the claimed time complexity. □

### Experimental Evaluation

In this section, we describe the methods used to generate the instance problems, assess the solutions generated by each algorithm, and compare the algorithms. This allows the assessment of the performance of each algorithm, Affine-DO in greater detail, and an evaluation of Affine-DO using exact solutions for trees with only 3 leaves.

### Data Sets

To generate the instance problems, We simulated a number of sequences using DAWG 1.1.1 [42] with insertions and deletions following a power law distribution. The simulations followed random binary trees of 50 leaves comprising all the combinations of the parameters listed in Table 1. These produced a total of 96,000 independent simulations. For each data set, we collected the true sequence assignment. This information allows the comparison of the cost calculated by Affine-DO with the cost implied by the true sequence of events. Our expectation was to produce costs lower than those using the true sequence assignment.

**Table 1:** Simulation parameters

**Parameter**	**Values Evaluated**
Substitution Rate	1*.*5
Average Branch Length	0*.*05,0*.*1,0*.*2,0*.*3,*∞*
Max. Gap	1,2,5,10,15
Root Sequence Length	70,100,150,200, 300,400,500,1000

All combinations of parameters were employed to generate the test data sets. The branch length variation equals the average branch length.

### Solution Assessment

The sequences assigned by the simulation can be far from the optimal solution. To evaluate Affine-DO, we used two algorithms: the standard Fixed States algorithm, which is known to be a 2-approximation, and the cost calculated by the solution of an LP instance of the problem. A good heuristic solution should always be located between these two bounds. As a comparison measure for each solution, the ratio between the solution cost and the LP bound was computed. The closer the ratio to 1*.*0, the better is the solution.

This form of evaluation has the main advantage (but also disadvantage), of being overly pessimistic. Most likely, the LP solution is unachievable, and therefore, the approximation ratio inferred for the solution produced by Affine-DO will most likely be an overestimate. To assess how over-negative the LP bound is, we produced 2100 random sequences divided in triplets of lengths between 70 and 1000. For each triplet, the Affine-DO, the LP bound, and the exact solution were computed. These three solutions were compared to provide an experimental overview of the potential performance of our algorithm. We selected random sequences because preliminary experiments showed evidence that these produce the most difficult instances for Affine-DO.

#### Algorithms compared

We implemented a number of algorithms to approximate the tree alignment problem. Our implementation can be divided in two groups: initial assignment, and iterative improvement.

##### Initial Assignment

includes the Fixed States (a stronger version of the Lifted Assignment [34,43]), Direct Optimization [15], and Affine-DO. Each of these algorithms starts with a function *χ* and creates a *χ*^*′*^ compatible with *χ*which is an instance solution. DO and Affine-DO have already been described. The Fixed States [43] is a simple algorithm were the interior vertices are optimally assigned one of the leaf sequences of the input tree, yielding a 2-approximation solution [34].

##### Iterative Improvement

**Figure 5:**
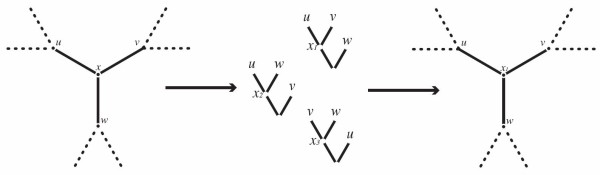
**Approximate DO.** An iteration of the approximated iterative improvement. To improve *x*, Affine-DO is used to produce *x*_1_, *x*_2_, and *x*_3_in the three possible rooted trees with leaves *u*,*v*, and *w*. If the best assignment *x*_1_yields better cost than the original *x*, then it is replaced, otherwise no change is made.

modifies an existing *χ*^*′*^by readjusting each interior vertex using its three neighbors. This procedure is repeated iteratively, until a (user provided) maximum number of iterations is reached, or no further tree cost improvements can be achieved. The adjustment itself can be done using an *approximated* or an *exact* three dimensional alignment, which we call the Approximate Iterative and Exact Iterative algorithms. Approximate Iterative (Figure 5), uses DO or Affine-DO (the selection depends on which kind of edit distance function is used) to solve the TAP on the three possible rooted trees formed by the three neighbors of the vertex used as leaves. The assignment yielding the best cost is selected as the new center. The exact three dimensional alignment has time complexity *O*(*n*^3^) [44]. Our implementation uses the low memory algorithms implemented by Powell [44], though they can be improved to *O*(*n*^2^) memory consumption [45].

We compared MSAM [32], Affine-DO, Approximate Iterative, Exact Iterative, and Fixed States, using a lower bound computed with an LP solution. We do not include DO in the comparisons because *it could not solve this problem*[46]. It is therefore impossible to compare it directly with our algorithm. GESTALT, SALSA, and PRODALI were unavailable, and so, could not be used in our comparative evaluation. TreeAlign did not produce a solution for the simulations within 48 hours of execution time, and therefore, was not included in the comparisons.

In total, more than 330,000 solutions were evaluated. We only present those results that show significant differences, and represent the overall patterns detected. The Exact Iterative algorithm was only evaluated for the short sequences (70 to 100 bases), due to the tremendous execution time it requires. Fixed States followed by iterative improvement is not included because its execution time is prohibitive for this number of tests (POY version 4 supports this type of analysis). Nevertheless, preliminary analyses showed that this combination of algorithms produce results in between Fixed States and Affine-DO, but not competitive with Affine-DO.

#### Algorithm Comparison

**Figure 6:**
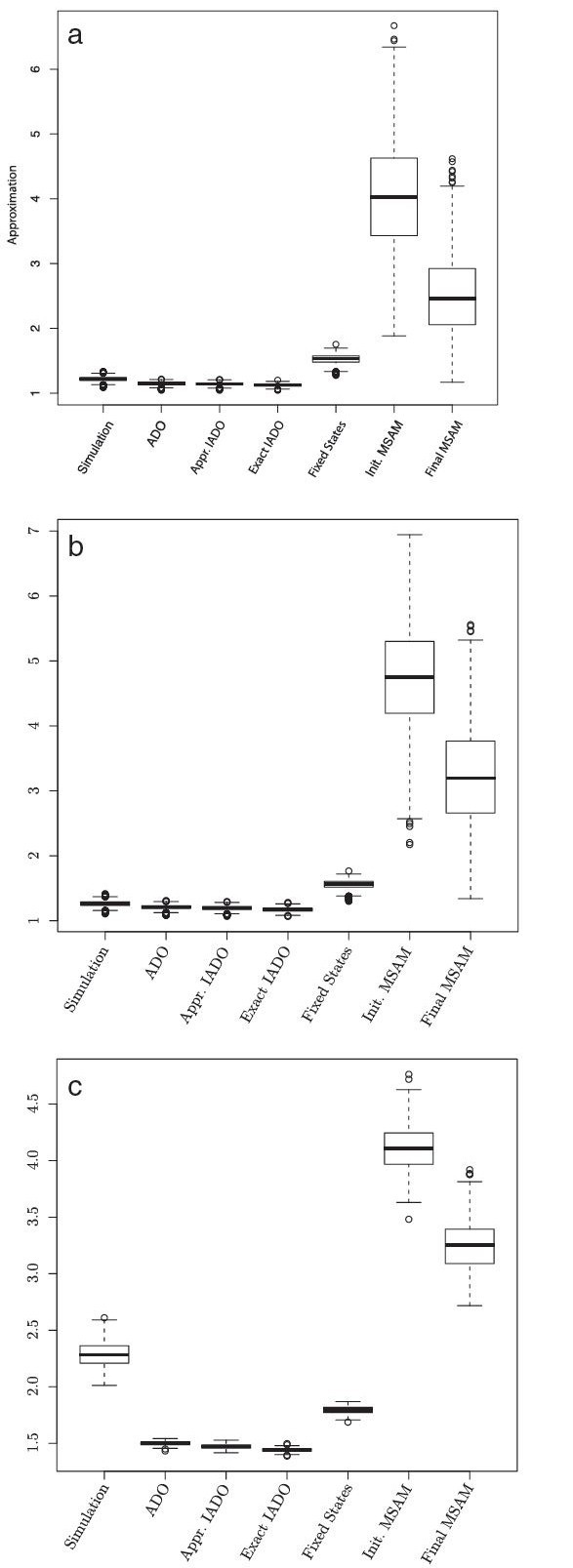
**Algorithm comparison.** General patterns observed in the approximation ratio of the different algorithms. Simulation is the simulated data, ADO is Affine-DO, Approx. and Exact IADO are the approximated and the exact iterative Affine-DO algorithms respectively, initial and final MSAM are the initial and final estimations of the MSAM algorithm. **a.** substitutions = 1, *a*=0, *b*=1, branch length=0*.*05. **b.** substitutions = 4, *a*=3, *b*=1, branch length=0*.*05. **c.** substitutions = 4, *a*=3, *b*=1, branch length=0*.*3.

The most important patterns observed between the evaluated algorithms are presented in Figure 6 and Table 2. In general, Affine-DO yields a better approximation than Fixed States. According to the density histograms (data not shown), the expected approximation ratio of 1.1 (versus 1.5 for Fixed States) in the best parameter combination, and 1.5 (versus 1.7) for the worst. Iterative improvement (both in exact and approximated forms) has a small overall impact in the approximation ratio (with a maximal decrease of 0*.*05 when compared with the solution inferred by Affine-DO alone). In all cases, Affine-DO found better solutions than the simulations (Table 2).

**Table 2:** Numerical comparison of a pair of parameter combinations that represents the variation observed between the different algorithms

**Subst.**	**Gap Op.**	**Branch Len.**	**Algorithm**	**Min.**	**Median**	**Max**
1	0	0*.*05	Simulated	1*.*088	1*.*218	1*.*337
			Fixed States	1*.*275	1*.*534	1*.*755
			ADO	1*.*044	1*.*148	1*.*215
			ADO + Iter.	1*.*044	1*.*123	1*.*202
1	0	0*.*3	Simulated	1*.*731	2*.*022	2*.*396
			Fixed States	1*.*621	1*.*725	1*.*816
			ADO	1*.*314	1*.*398	1*.*453
			ADO + Iter.	1*.*300	1*.*377	1*.*393
4	3	0*.*05	Simulated	1*.*108	1*.*262	1*.*415
			Fixed States	1*.*302	1*.*557	1*.*766
			ADO	1*.*084	1*.*208	1*.*312
			ADO + Iter.	1*.*067	1*.*171	1*.*283
4	3	0*.*3	Simulated	2*.*012	2*.*284	2*.*611
			Fixed States	1*.*688	1*.*795	1*.*868
			ADO	1*.*433	1*.*500	1*.*542
			ADO + Iter.	1*.*388	1*.*442	1*.*453

Each individual indel has cost 1.

Although the combination of Affine-DO and Iterative improvement produces better solutions, its execution time is dramatically higher. In the current implementation, running on a 3.0 Ghz, 64 bit Intel Xeon 5160 CPU with 32 GB of RAM, Affine-DO evaluates each tree in less than 1 second in the worst case, while Affine-DO + Iterative improvement may take more than 1 hour per tree. For this reason, Affine-DO is well suited for heuristics that require a very large number of tree evaluations such as the GTAP, where millions of trees are evaluated during a heuristic search.

#### Approximation of Affine-DO

**Figure 7:**
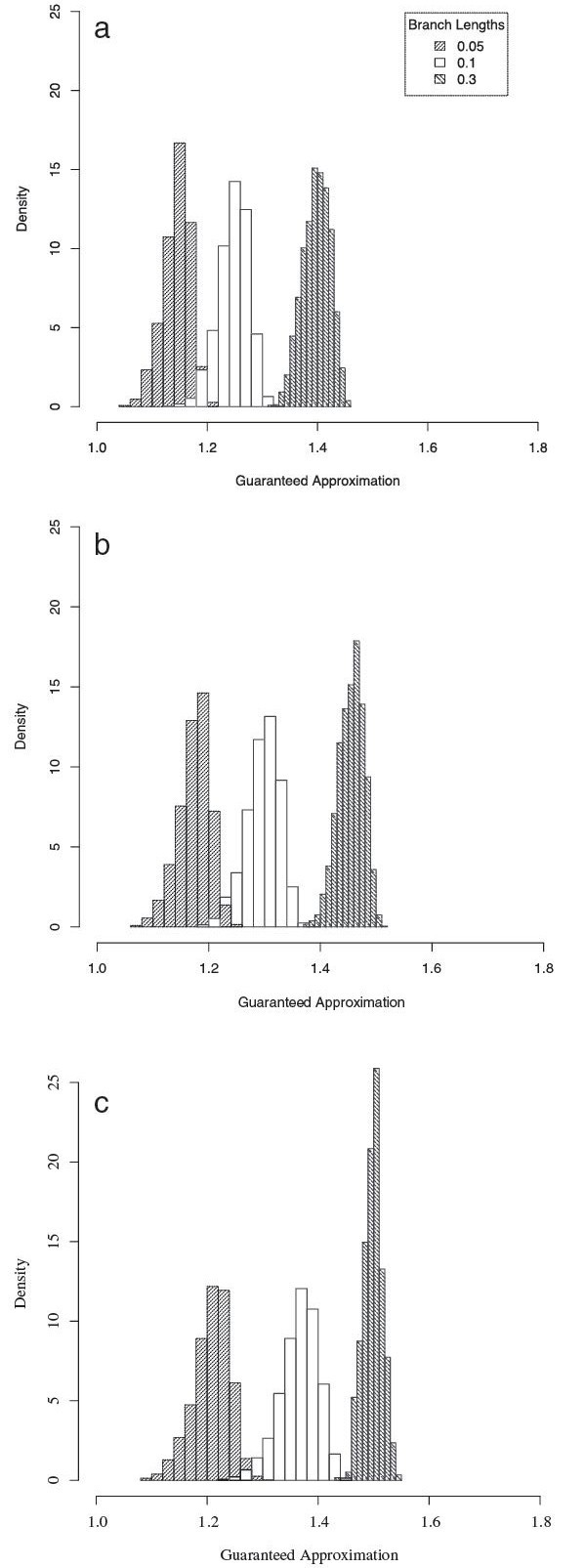
**Affine-DO vs.** Theoretical LP bound. Guaranteed approximation ratio of Affine-DO compared with the theoretical LP bound, for different cost and sequence generation parameters. **a.** substitutions = 1, *a*=0, *b*=1. **b.** substitutions = 2, *a*=1, *b*=1. **c.** substitutions = 4, *a*=1, *b*=3.

Figure 7 shows the density histogram of the guaranteed approximation of the Affine-DO algorithm when compared with the LP theoretical solution for a representative set of parameters. The results show that Affine-DO has a guaranteed approximation of less than 60% in every case.

**Figure 8:**
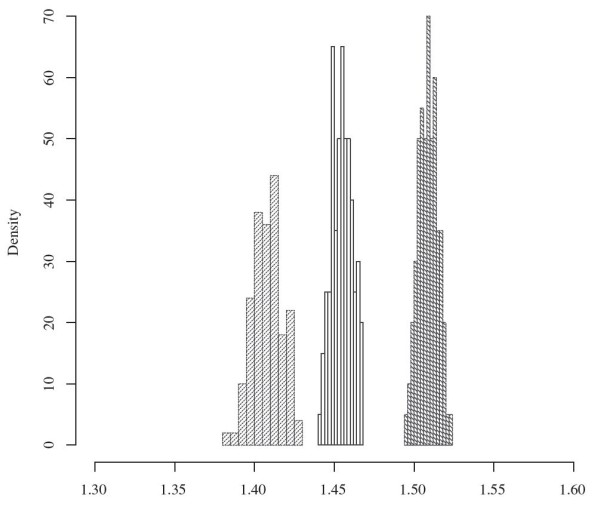
**Affine-DO vs** Theoretical LP bound with random sequences. Guaranteed approximation of Affine-DO for random sequences. In the left substitutions=1, *a*=0, *b*=2, in the center substitutions=1, *a*=0, *b*=1, and in the right substitutions=2, *a*=1, *b*=1. These are representative of the distributions observed in the experiments.

Typically, the larger the sequence divergence, the larger is the approximation degree of Affine-DO. The same pattern is observed for larger *a*. To test an extreme case, were the branch length is maximal, we evaluated the behavior of random sequences in the same set of trees. Figure 8 shows the results of this experiment.

The worst case is observed with an average approximation slightly over 1*.*5. This variation, however, could have been caused by a more relaxed LP bound, which could be producing an overly pessimistic evaluation of the algorithm. To assess the importance of this factor, we evaluated its tightness experimentally.

#### Comparison with an exact solution

**Figure 9:**
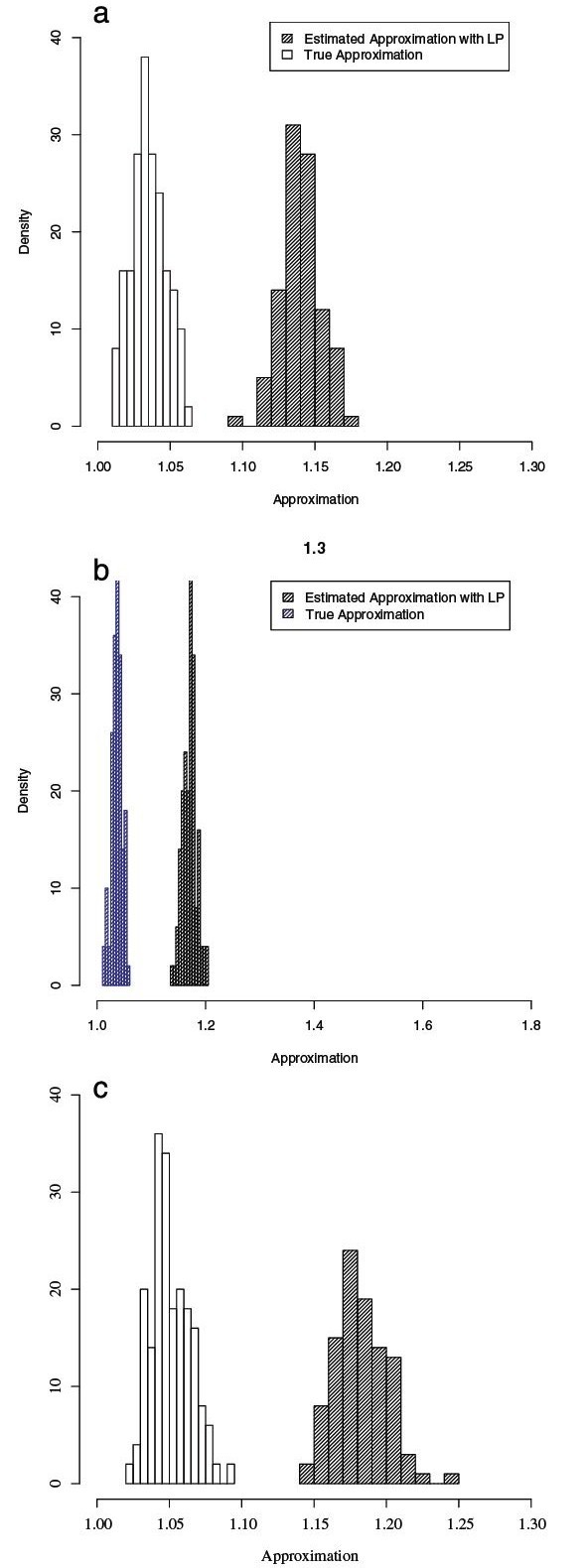
**Affine-DO vs.** exact solution. Tightness of the Affine-DO solution according to the LP bound compared to the exact approximation. Observe that even for a very small data set, the LP bound is not realistic, and Affine-DO is close to the optimal solution. **a.** substitutions = 1, *a*=0, *b*=1. **b.** substitutions = 2, *a*=1, *b*=1. **c.** substitutions = 4, *a*=1, *b*=3.

To assess Affine-DO and the tightness of the LP bound, we computed the exact solution for 700 unrooted trees consisting of 3 leaves with random sequences assigned to their leaves, under all the parameter sets tested. Figure 9 shows the density histograms for the results obtained.

Note that the LP-inferred bound is overly negative even for these small test data sets, with the inferred approximation expected at around 1.15, while in reality Affine-DO finds solutions that are expected to approximate within 1.05 of the optimal solution, a 10% difference for trees consisting of only 3 sequences.

## Conclusions

We have presented a novel algorithm that we have called Affine-DO for the TAP under affine gap costs. Our experimental evaluation, the largest performed for this kind of problem, shows that Affine-DO performs better than Fixed States. However, we observed that the LP bound is too pessimistic, producing unfeasible solutions 10% worse, even for the smallest non-trivial tree consisting of 3 leaves. Based on these observations, we believe that Affine-DO is producing near-optimal solutions, with approximations within 10% for sequences with small divergence, and within 30% for random sequences, for which Affine-DO produced the worst solutions.

Affine-DO is well suited for the GTAP under affine sequence edit distances, and yields significantly better results when augmented with iterative methods. The main open question is whether or not there exists a guaranteed bound for DO or Affine-DO. Then, if the answer is positive, whether or not it is possible to improve the PTAS using these ideas. Additionally, many of these ideas can be applied for true simultaneous tree and alignment estimation under other optimality criteria such as ML and MAP. Their use under these different optimality criteria remains to be explored.

## Competing interests

The authors declare that they have no competing interests.

## Authors’ contributions

WW defined the Fixed States and DO algorithms. AV developed Affine-DO and performed all analyses under supervision of WW. AV and WW wrote and revised the manuscript. Both authors read and approved the final manuscript for publication.
